# Feeling pain from a rubber hand: Nociceptive drift in the rubber hand illusion

**DOI:** 10.1016/j.isci.2025.112690

**Published:** 2025-05-19

**Authors:** Sara Coppi, Karin B. Jensen, H. Henrik Ehrsson

**Affiliations:** 1 Department of Neuroscience, Karolinska Institutet, Stockholm, Sweden; 2 Department of Clinical Neuroscience, Karolinska Institutet, Stockholm, Sweden

**Keywords:** Behavioral neuroscience, Cognitive neuroscience

## Abstract

The sense of body ownership refers to the perceptual experience of one’s body as one’s own and can be studied using the rubber hand illusion (RHI). Body ownership relies on multisensory integration, although the relative contributions of different sensory modalities remain unclear. We investigated the role of nociception in body ownership using the nociceptive RHI (N-RHI), a version of the RHI triggered by noxious laser stimulation. Specifically, we assessed whether the N-RHI is as strong as the classic tactile RHI and whether both illusions induce a shift in perceived pain location toward the rubber hand (“nociceptive drift”). Our results reveal similarly strong questionnaire responses and proprioceptive drift in both illusions compared with the control conditions and show that both the tactile RHI and the N-RHI result in a significant nociceptive drift. These findings underscore the role of nociception in body ownership and highlight the multisensory nature of pain localization.

## Highlights

• Visual and nociceptive signals integrate, like visuotactile cues in the rubber hand illusion
• A new task, nociceptive drift, tracks shifts in perceived pain location
• Pain location shifts toward a rubber hand experienced as part of one’s own body

## Graphical abstract

**Figure:**
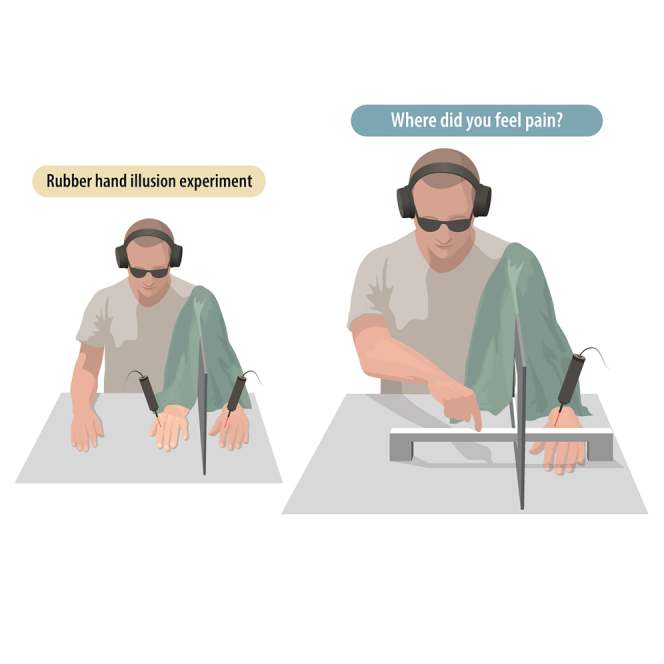

## Introduction

Body ownership is the perceptual feeling that our body is our own.^1^^,^^2^ This perception of one’s own body cannot be reduced to unisensory somatosensory processing, such as touch or proprioception, but instead arises from the integration of sensory information across multiple modalities to form a coherent perception of one’s body in space.^3^^,^^4^ The rubber hand illusion (RHI)^5^ is a well-known bodily illusion and is the most commonly used experimental paradigm to study body ownership in healthy individuals.^1^^,^^3^^,^^4^^,^^5^^,^^6^^,^^7^ In this illusion, participants experience somatosensory sensations originating from a rubber hand, which they perceive as their own after a period of synchronous stroking of the fake hand and the participant’s real hand that is hidden from view. Research on body ownership using the RHI and similar body ownership illusions has demonstrated that these illusions follow multisensory integration principles.^1^^,^^3^^,^^4^^,^^5^^,^^8^^,^^9^ Specifically, the temporal and spatial congruence of sensory signals across different modalities is critical for eliciting the RHI and altering body ownership perception. While early research focused on correlated visual and tactile signals in the RHI, studies over the past decade have highlighted the contributions of many different sensory channels. For example, changes in body ownership perception in RHI paradigms can arise from correlations among vision, touch, and proprioception^8^^,^^10^; vision and balance^11^; vision and proprioception^12^^,^^13^; vision, touch, proprioception, and thermosensation^14^; vision, touch, proprioception, and audition^15^; and vision, touch, proprioception, and nociception.^16^^,^^17^ However, among the bodily senses, the roles of nociception and pain in body ownership perception remain under-investigated. This lack of research is surprising given that pain plays crucial roles in survival and self-preservation,^18^^,^^19^ has a fundamental link to self-experience,^20^ and is “intrinsically motivating exclusively for body-directed behavior.”^21^ Intuitively, it is plausible that pain and body ownership should be related, as they are both fundamental embodied experiences linked to the self.

Recently, we introduced an RHI paradigm showing that the RHI can be elicited by correlated visual and “pure” nociceptive input (herein “N-RHI”^22^). In the N-RHI, the real hand is hidden behind a screen, as in the tactile RHI. However, instead of tactile stroking, nociceptive laser stimuli (Nd:YAP laser; see STAR Methods) are applied to the real hand while synchronized red laser lights (LEDs) are projected onto the rubber hand, matching the location and timing of the stimuli applied to both hands. Critically, the Nd:YAP laser activates C and Aδ nociceptors^23^^,^^24^ in the skin without activating the tactile A*β* mechanoreceptors,^25^ enabling the testing of nociceptive contributions without contamination from tactile inputs. We found that the N-RHI follows temporal and spatial congruency principles similar to those described for the tactile RHI.^1^^,^^26^^,^^27^ Specifically, Coppi et al.^22^ reported that, compared with asynchronous and spatially incongruent visual nociceptive stimulation, synchronous and spatially congruent visual nociceptive stimulation increased ownership ratings and proprioceptive drift toward the rubber hand. The N-RHI is important in research because it demonstrates that signals from the C and Aδ nociceptors are sufficient to integrate with visual feedback and update the sense of body ownership. Thus, pain not only is an alarm signal for potential or actual tissue damage but also plays a role in shaping bodily perception.

However, two important questions regarding the N-RHI remain unresolved and warrant further investigation. First, does the N-RHI elicit a full illusion or only a weaker version of the classic tactile RHI? In our previous study, we did not directly compare the N-RHI with the tactile RHI within the same experiment, leaving this question unanswered. For the same reason, it is unclear whether the effects of multisensory congruence and incongruence are comparable between the two illusions, which would be expected if the illusions rely on similar multisensory processes. However, Coppi et al.^22^ reported that the number of participants affirming the illusion and the group median scores of illusion strength were somewhat lower than those reported in previous RHI studies. This difference may suggest that nociception contributes less to body ownership than touch does; however, it could also reflect the brief duration of the nociceptive laser stimulus (7 ms), which is much shorter than the relatively long tactile brushstrokes used in most RHI experiments (several hundred milliseconds to a second). Consequently, the shorter sensory stimulation in the N-RHI paradigm may provide less strongly correlated visual nociceptive input, reducing the strength of the illusion. Thus, determining whether congruent visual nociceptive input produces RHI effects comparable to those elicited by congruent visuotactile stimuli is critical to understanding whether similar multisensory processes underlie both illusions. In the present study, we addressed this question in two experiments examining whether the N-RHI is comparable in strength to the tactile RHI on the basis of classic RHI measures: questionnaire ratings^5^ (experiment 1) and proprioceptive drift^5^^,^^28^ (experiment 2; see below).

Second, if nociception contributes to the RHI in a similar manner to other somatosensory submodalities—and, by extension, to multisensory bodily awareness—a drift in the perceived location of a painful stimulus on the real hand toward the rubber hand after experiencing the illusion would be expected. This “nociceptive drift” would parallel the proprioceptive drift that occurs during the RHI,^5^^,^^28^^,^^29^^,^^30^ where people judge the location of their hidden hand to be closer to the rubber hand than it actually is. Proprioceptive drift is believed to reflect the multisensory nature of limb localization,^31^ which is influenced by body ownership through the updating of hand position estimates. The demonstration of a “nociceptive drift” after both nociceptive and tactile RHIs would underscore the multisensory nature of pain localization and its link to body ownership. Additionally, this drift would indicate that the visual‒somatic spatial conflict in the RHI is resolved not only by proprioceptive recalibration but also by pain recalibration, suggesting that the intersensory recalibration processes associated with body ownership involve multiple sensory modalities. In this study, we addressed this question in a third experiment by introducing a novel “nociceptive drift test” to examine whether the N-RHI and tactile RHI are both associated with significant nociceptive drift toward the rubber hand (experiment 3).

### Present study

**Figure 1:**
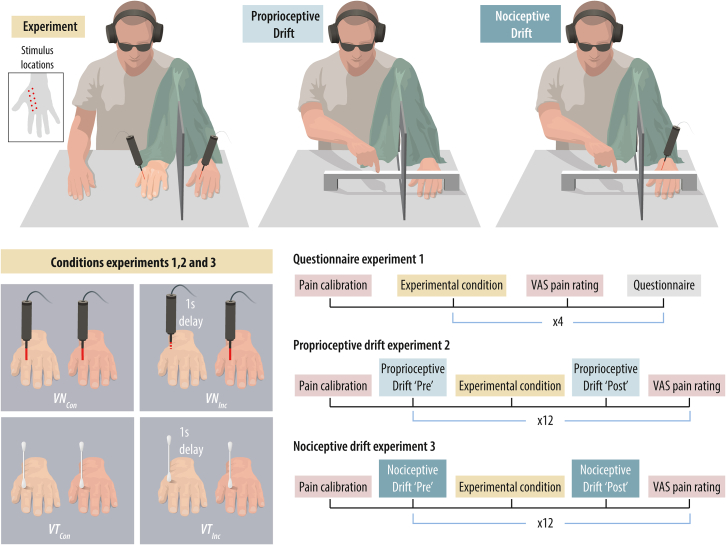
Experimental paradigm: tactile and nociceptive rubber hand illusions The top-left, top-middle, and top-right panels depict the experimental setups. The bottom-left panel illustrates the four conditions used across all experiments: visuonociceptive congruent (VN_Con_, top-left), visuonociceptive incongruent (VN_Inc_, top-right), visuotactile congruent (VT_Con_, bottom-left), and visuotactile incongruent (VT_Inc_, bottom-right). The bottom-right panel shows the procedural timeline for each experiment.

The three experiments involved separate groups of healthy participants (see Table 1) and used the same four experimental conditions (see Figure 1). As in Coppi et al.,^22^ all nociceptive stimuli were generated by a Nd:YAP laser to selectively activate C and Aδ nociceptors without coactivating tactile mechanoreceptors, thus providing “pure” nociceptive stimulation. To induce the N-RHI, we used the visual nociceptive congruent condition (VN_Con_), in which synchronous visual and nociceptive stimulation was applied to the rubber hand (in view) and the participant’s real hand (hidden) at corresponding sites. The nociceptive stimulus was a painful Nd:YAP laser pulse (7 ms duration), whereas the visual stimulus was a small red dot generated by a diode laser (130 ms duration). The visual nociceptive incongruent condition (VN_Inc_) served as the critical control; in this condition, a 1-s delay was introduced between the visual and nociceptive stimuli, and different sites on the hands were stimulated. To induce the tactile RHI, we used the visuotactile congruent condition (VT_Con_), in which the rubber hand and the real hand were tapped synchronously at corresponding sites with two cotton swabs (approximate tap duration: 130 ms). The visuotactile incongruent condition (VT_Inc_) served as the control, introducing a 1-s delay between the visual and tactile stimuli while stimulating different sites on the two hands (see Figure 1).

**Table 1:** Descriptive statistics of participant characteristics and calibration data

Experiment	Measurement	Blocks (N)	Participants	Fluence mJ/mm^2^	VAS during pain calibration
1	Questionnaire	4: 2 visuo-tactile & 2 visuo-nociceptive	*N* = 30 (8 M–22 F) M_age_ = 25.3 SD_age_ = 4.28 28 right-handed	M = 71.7 SD = 21.3 Min = 26 Max = 123	M = 22.7 SD = 4.85 Min = 11.5 Max = 34.9
1	Illusion onset	3 visuo-tactile	*N* = 19 (5 M–14 F) M_age_ = 25.74 SD_age_ = 4.62 17 right-handed	NA	NA
1	Illusion onset	3 visuo-nociceptive	*N* = 16 (5 M–11 F) M_age_ = 24.75 SD_age_ = 5.08 15 right-handed	M = 72.31 SD = 20.81 Min = 39 Max = 110.5	M = 21.23 SD = 4.61 Min = 11.5 Max = 30
2	Proprioceptive Drift	12: 6 visuo-nociceptive & 6 visuo-tactile	*N* = 30 (16 M–14 F) M_age_ = 26.1 SD_age_ = 5.87 28 right-handed	M = 58.5 SD = 15.4 Min = 26 Max = 84.5	M = 22.2 SD = 3.52 Min = 15.9 Max = 30.7
3	Nociceptive Drift	12: 6 visuo-nociceptive & 6 visuo-tactile	30 = 15 M–15 F M_age_ = 25.2 SD_age_ = 5.52 29 right-handed	M = 59.4 SD = 20.7 Min = 26 Max = 97.5	M = 23.1 SD = 4.46 Min = 18.1 Max = 35.4

The table presents the descriptive statistics of the participants, the fluence energy used in each experiment, and the pain ratings reported during the calibration phase. Pain was measured using a visual analog scale (VAS) ranging from 0 (‘no pain at all’) to 100 (‘the worst imaginable pain). The values shown include data from all participants prior to outlier removal. In Experiment 1, the same sample population was used for both the Questionnaire and the Illusion Onset tasks. However, we report them separately, as only participants who reported experiencing ownership of the rubber hand in the questionnaire proceeded to perform the Illusion Onset task. No laser stimulation was applied during the visuo-tactile trials.

In experiment 1, we assessed the illusion using a questionnaire in which participants rated their agreement or disagreement with four statements related to their subjective experience of the illusion (Table 2). Responses were recorded on a Likert scale ranging from −3 (“completely disagree”) to +3 (“completely agree”). For both RHI variants, we expected significantly greater illusion ratings in the congruent conditions than in the incongruent conditions. Additionally, as an exploratory measure, we recorded the illusion onset time (i.e., the time in seconds until the participants first experienced the illusion) after the questionnaire was completed.

**Table 2:** Questionnaire

Statement number	Statement	Statement class
S1. Hand Ownership	‘I felt as if the rubber hand were my hand’	*Illusion* (visuo-nociceptive/tactile-proprioceptive integration)
S2. Hand ownership	‘It felt as if the rubber hand were part of my body’	*Illusion* (visuo-nociceptive/tactile-proprioceptive integration)
S3. Control hand	‘It seemed as if I might have more than one left hand or arm’	*Control*
S4. Control hand	‘It seemed as if my real hand was larger than normal’	*Control*
S5. Referral of pain	‘It seemed as if I were feeling the pain in the location where I saw the light on the rubber hand’	*Illusion* (visuo-nociceptive integration)
S5. Referral of touch	‘It seemed as if I were feeling the touch in the location where I saw the cotton swab touching the rubber hand’	*Illusion* (visuo-tactile integration)
S6. Referral of pain	‘It felt as if the painful sensation I felt was caused by the laser light on the rubber hand’	*Illusion* (visuo-nociceptive integration)
S6. Referral of touch	‘It felt as if the tactile sensation I felt was caused by the cotton swab on the rubber hand’	*Illusion* (visuo-tactile integration)
S7. Control	‘It seemed as if the pain I felt came from somewhere between my own hand and the rubber hand’	*Control* (visuo-nociceptive integration)
S7. Control	‘It seemed as if the touch I felt came from somewhere between my own hand and the rubber hand’	*Control* (visuo-tactile integration)
S8. Control	‘It seemed as if my real hand was cold’	*Control*

The table shows the statements presented in the questionnaire. The statements were answered on a 7-point Likert scale ranging from −3 (strongly disagree) to +3 (strongly agree).

In experiment 2, we assessed the N-RHI and tactile RHI using the proprioceptive drift task.^5^^,^^22^^,^^28^ In this task, participants used their right index finger to indicate the perceived location of their left index finger before and after exposure to each of the four conditions described above. The difference between pre- and postexposure indications represents “proprioceptive drift”, reflecting the extent to which participants incorrectly judge the location of their left index finger to be closer to the rubber hand. Previous studies have shown that proprioceptive drift is greater after experiencing the N-RHI or tactile RHI than in their respective control conditions.^22^^,^^28^^,^^30^

In experiment 3, we introduced the new nociceptive drift task, in which participants, with their eyes closed, indicated the perceived location of a painful nociceptive stimulus delivered to their hidden hand. Immediately before and immediately after exposure to each of the four experimental conditions, a nociceptive laser-induced stimulus was delivered to the first knuckle of the participants’ left index finger. Each time, the participant used their right index finger to indicate where they perceived the painful stimulation on their hidden left hand. The difference between pre- and postexposure indications corresponds to “nociceptive drift”. We expected greater nociceptive drift after experiencing either version of the RHI than in the control conditions—i.e., participants would incorrectly judge the location of painful stimuli to be closer to the rubber hand after the illusion.

Beyond these specific hypotheses, our overarching question across the three experiments was whether the N-RHI was as strong as or weaker than the tactile RHI. To address this question, we compared the congruence-specific effects of the two illusions (VN_Con_ minus VN_Inc_ compared to VT_Con_ minus VT_Inc_) on questionnaire ratings (experiment 1), proprioceptive drift (experiment 2), and nociceptive drift (experiment 3). While we did not have a strong hypothesis beyond expecting robust RHI elicitation with both variants, these comparisons allowed us to assess potential differences in illusion strength between the two modalities while keeping all other factors equivalent.

## Results

### Experiment 1

#### Questionnaire results

**Figure 2:**
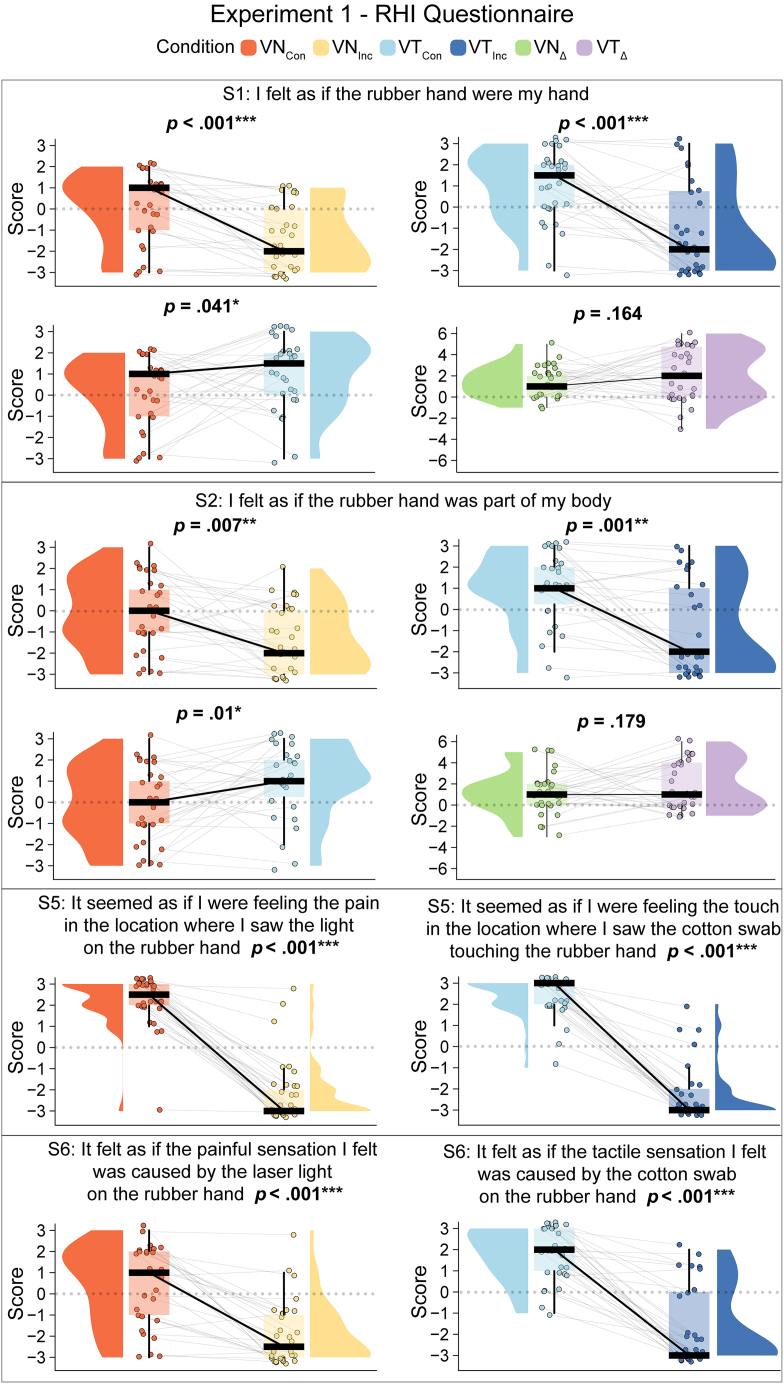
Paired comparisons in experiment 1 Raincloud plots illustrate the individual data points and distributions of illusion-related questionnaire responses from experiment 1 (*N* = 30). Each plot also represents a boxplot summarizing the median and quartiles. Comparisons are shown for VN_Con_ vs. VN_Inc_, VT_Con_ vs. VT_Inc_, VN_Con_ vs. VT_Con_, and VN_Δ_ vs. VT_Δ_. Responses were rated on a Likert scale ranging from −3 (strongly disagree) to +3 (strongly agree). Note: *p* < 0.05 (∗), *p* < 0.01 (∗∗), *p* < 0.001 (∗∗∗); reported *p*-values are uncorrected. Δ denotes the difference between congruent and incongruent conditions.

As expected, all four illusion statements, including two statements explicitly referring to illusory ownership of the rubber hand (S1, S2) and two statements referring to sensing pain from the rubber hand (S5, S6) (i.e., “illusory referral of pain”) were rated statistically higher in the *VN*_*Con*_ condition than in the *VN*_*Inc*_ (S1: *V* = 266, 95% CI = [1.5, 2.5], *p* < 0.001, BF_10_ > 1000, *r*_*C*_ = 0.93; S2: *V* = 242.5, 95% CI = [0.5, 2], *p* = 0.007, BF_10_ = 9.53, *r*_*C*_ = 0.62; S5: *V* = 406, 95% CI = [4, 5], *p* < 0.001, BF_10_ > 1000, *r*_*C*_ = 1; S6: *V* = 274, 95% CI = [2, 3.5], *p* < 0.001, BF_10_ > 1000, *r*_*C*_ = 0.99) (see Figure 2).

Similarly, all illusion statements, including both hand-ownership (S1, S2) statements and touch-referral statements (S5, S6), were rated statistically higher in the *VT*_*Con*_ condition than in the *VT*_*Inc*_ condition (S1: *V* = 235.5, 95% CI = [2,4.5], *p* < 0.001, BF_10_ > 100, *r*_*C*_ = 0.86; S2: *V* = 248.5, 95% CI = [1.5, 4], *p* = 0.001, BF_10_ > 100, *r*_*C*_ = 0.8; S5: *V* = 465, 95% CI = [4, 5.5], *p* < 0.001, BF_10_ > 1000, *r*_*C*_ = 1; S6: *V* = 406, 95% CI = [3, 4.5], *p* < 0.001, BF_10_ > 1000, *r*_*C*_ = 1) (see Figure 2). Therefore, the questionnaire data in the current study replicated both the tactile RHI and the N-RHI.

In the comparison of the two variants of the RHI, we found that hand ownership scores in S1 were statistically significantly higher in the *VT*_*Con*_ condition than in the *VN*_*Con*_ condition (S1: *V* = 238, 95% CI = [0, 2.5], *p* = 0.041, BF_10_ = 1.3, *r*_*C*_ = 0.47). Similarly, the hand ownership scores in S2 were statistically significantly higher in the *VT*_*Con*_ condition than in the *VN*_*Con*_ condition (S2: *V* = 276, 95% CI = [0.5, 2.5], *p* = 0.01, BF_10_ = 4.47, *r*_*C*_ = 0.57) (see Figure 2).

Next, we compared the illusion strength between the two variants of the RHI by comparing the ratings on the illusion statements S1 and S2 in each illusion condition to their respective incongruent control conditions and then comparing the difference in these difference measures across illusions (i.e., *VN*_*Con*_ minus *VN*_*Inc*_ versus *VT*_*Con*_ minus *VT*_*Inc*_). The advantage of this approach is that it controls for possible nonspecific effects of stimulus modality on illusion ratings. Additionally, difference scores are less susceptible to cognitive bias than raw questionnaire ratings from an individual condition.^32^^,^^33^ Importantly, no statistically significant difference between the two difference measures was found for either S1 or S2 (S1: *V* = 121, 95% CI = [−2.5, 0.5], *p* = 0.164, BF_01_ = 2.71, *r*_*C*_ = −0.311; S2: *V* = 123, 95% CI = [−3, 0.5], *p* = 0.179, BF_01_ = 2.4, *r*_*C*_ = −0.299).

Additionally, we descriptively compared the pain referral and touch referral statements across RHIs via a post hoc approach. However, these results should be interpreted with caution, as the statements used slightly different wording. Specifically, we compared the difference in touch referral (*VT*_*Con*_ minus *VT*_*Inc*_) in the tactile RHI with the difference in pain referral (*VN*_*Con*_ minus *VN*_*Inc*_) in the nociceptive RHI for both S5 and S6. No statistically significant difference was found between touch referral and pain referral for S5 (*V* = 57.5, 95% CI = [−2, 1], *p* = 0.364, BF_01_ = 3.63, *r*_*C*_ = −0.248). However, for S6, a statistically significant difference was observed in favor of touch referral (*V* = 103, 95% CI = [−2.5, 0], *p* = 0.037, BF_10_ = 2.09, *r*_*C*_ = −0.455).

In an exploratory post hoc analysis, we found no significant correlation between the subjective strength of visuotactile and visual nociceptive illusions (i.e., *VT*_*Con*_ minus *VT*_*Inc*_ was not correlated with *VN*_*Con*_ minus *VN*_*Inc*_) (S1: *r*_*S*_ = −0.24, *p* = 0.2; S2: *r*_*S*_ = −0.08, *p* = 0.66) or when analyzing only the congruent conditions (*VN*_*Con*_ *and VT*_*Con*_) (S1: *r*_*S*_ = −0.03, *p* = 0.89; S2: *r*_*S*_ = 0.14, *p* = 0.48). Thus, a participant’s susceptibility to the tactile RHI did not appear to predict their susceptibility to the N-RHI, which is consistent with the belief that touch and nociception are distinct somatosensory submodalities and that individual differences in sensory processing within these systems may vary.^34^

#### Illusion onset results

One participant was identified as an outlier in the VT_Con_ condition and was therefore excluded from the analysis. We examined the onset times of the tactile RHI in 18 participants who reported illusory ownership of the rubber hand (i.e., those who rated statement S1 ≥ 1 after the *VT*_*Con*_ condition). The average illusion onset time for these participants was M = 14.57 s (SD = 12.14). Similarly, we analyzed the N-RHI onset time in 15 participants who responded ≥1 on statement S1 after the *VN*_*Con*_ condition. The average illusion onset time for these participants was M = 18.36 s (SD = 9.68). Statistically, the onset times of the two illusions did not differ (*W* = 94, 95% CI = [−13.36; 2.26], *p* = 0.145, BF_01_ = 2.08, *r*_*G*_ = −0.3).

**Figure 3:**
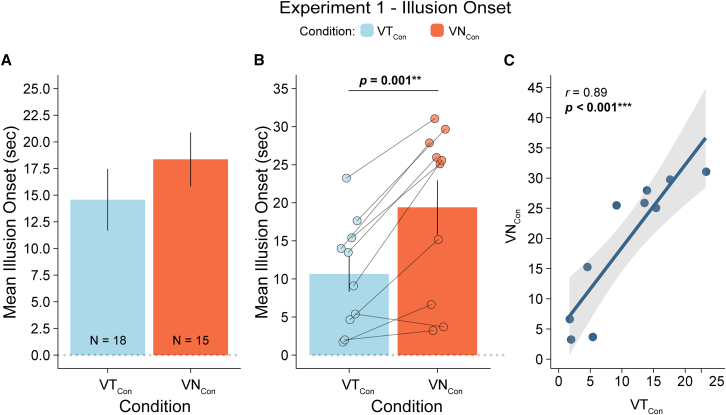
Illusion onsets in experiment 1 (A) Bar plots display the mean illusion onset times (in seconds) for both the classical Rubber Hand Illusion (RHI) and the nociceptive RHI (N-RHI). Data are represented as mean ± SE. (B) Bar plots present the average illusion onset times for participants who experienced both RHI and N-RHI (*N* = 10). Data are represented as mean ± SE. (C) Correlation plot showing the relationship between illusion onset times in the classical and nociceptive RHI conditions for participants who experienced both illusions (*N* = 10). Note: *p* < 0.05 (∗), *p* < 0.01 (∗∗), *p* < 0.001 (∗∗∗); reported *p*-values are uncorrected.

In contrast, participants who rated statement S1 ≥ 1 for both *VT*_*Con*_ and *VN*_*Con*_ (*n* = 10) required significantly less time to experience the illusion in the *VT*_*Con*_ condition than in the *VN*_*Con*_ condition (*t(9)* = 4.82, 95% CI = [4.63; 12.84], *p* = 0.001, BF_10_ = 42.65, *d*_*z*_ = 1.52). The average illusion onset in the *VT*_*Con*_ condition for these 10 subjects was M = 10.66 s (SD = 7.23), whereas the average onset in the *VN*_*Con*_ condition was M = 19.39 s (SD = 11.13). For these participants, the illusion onset times in the congruent conditions were positively correlated (*r(8)* = 0.89, 95% CI = [0.59; 0.97], *p* = 0.0005, BF_10_ = 19.78) (see Figure 3).

#### VAS results

VAS ratings did not differ significantly across conditions, blocks, or sexes (all *p* > 0.185). See Table S1.

### Experiment 2

#### Proprioceptive drift results

One participant was identified as an outlier in the VN_Con_ and VT_Con_ conditions and was therefore removed from the analysis, leaving 29 datasets. After outlier removal, the data were normally distributed (Shapiro‒Wilk *p value* for each condition >0.23) and met the assumption of homogeneity of variance (Levene’s test *p value* = 0.18). Thus, we conducted a 2 × 2 repeated-measures ANOVA, which revealed a significant main effect of congruence (*F*(1,28) = 11.598, *p* = 0.002, *η*_*p*_^2^ = 0.293) and modality (*F*(1,28) = 8.579, *p* = 0.007, *η*_*p*_^2^ = 0.235) but no interaction effect (*F*(1,28) = 0.26, *p* = 0.614, *η*_*p*_^2^ = 0.009).

**Figure 4:**
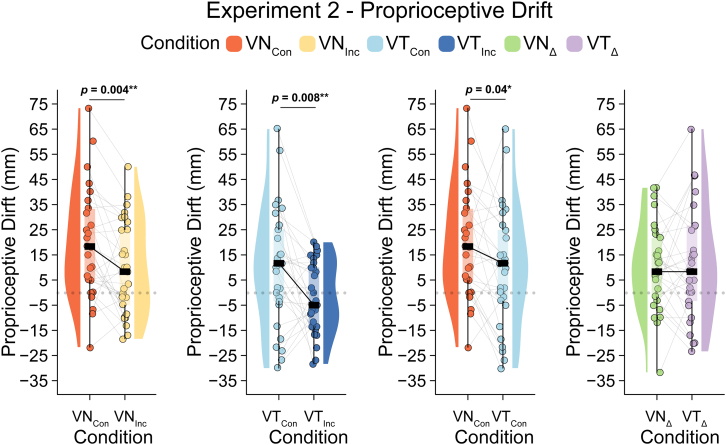
Proprioceptive drift in experiment 2 Raincloud plots illustrate the individual data points and distributions for the key condition comparisons. Data are represented as medians and IQR. Note: *p* < 0.05 (∗), *p* < 0.01 (∗∗), *p* < 0.001 (∗∗∗); reported *p*-values are uncorrected. Δ denotes the difference between congruent and incongruent conditions.

We further analyzed the significant main effect of congruence with two planned pairwise comparisons based on strong hypotheses. We found that proprioceptive drift toward the rubber hand was significantly greater after the *VT*_*Con*_ condition (M = 9.94, SD = 23.49) than after the *VT*_*Inc*_ condition (M = −2.07, SD = 14.03) (*t(28)* = 2.84, 95% CI = [3.33, 20.69], *p* = 0.008, BF_10_ = 5.27, *d*_*z*_ = 0.53) (Figure 4). Similarly, proprioceptive drift was significantly greater after the *VN*_*Con*_ condition (M = 19.6, SD = 21.82) than after the *VN*_*Inc*_ condition (M = 9.54, SD = 18.53) (*t(28)* = 3.12, 95% CI = [3.45, 16.66], *p* = 0.004, BF_10_ = 9.61, *d*_*z*_ = 0.58). Thus, the proprioceptive drift results support the successful induction of the illusion in both variants. See Figure 4.

While the nonsignificant interaction effect when comparing the illusion and respective control conditions argues against differences in RHI strength between the tactile and nociceptive variants, we examined the unexpected significant main effect of modality using pairwise tests. We found that proprioceptive drift was significantly greater in the *VN*_*Con*_ condition than in the *VT*_*Con*_ condition (*t(28)* = 2.19, 95% CI = [0.63, 18.68], *p* = 0.04, BF_10_ = 1.55, *d*_*z*_ = 0.41) and in the *VN*_*Inc*_ condition than in the *VT*_*Inc*_ condition (*t(28)* = 3.07, 95% CI = [3.87, 19.35], *p* = 0.005, BF_10_ = 8.66, *d*_*z*_ = 0.57) (see Table 3; Figure 4).

**Table 3:** Descriptive statistics for Experiments 1, 2, and 3

	VN_Con_ *Mean (SD)*	VN_Con_ *Median (1Q∼3Q)*	VN_Inc_ *Mean (SD)*	VN_Inc_ *Median (1Q∼3Q)*	VT_Con_ *Mean (SD)*	VT_Con_ *Median (1Q∼3Q)*	VT_Inc_ *Mean (SD)*	VT_Inc_ *Median (1Q∼3Q)*
Questionnaire – S1	0.1 (1.67)	1 (−1 ∼ 1)	−1.33 (1.52)	−2 (−3 ∼ 0)	1 (1.72)	1.5 (0∼2)	−1.13 (2.13)	−2 (−3 ∼ 0.75)
Questionnaire – S2	−0.17 (1.78)	0 (−1 ∼ 1)	−1.3 (1.56)	−2 (−3 ∼ 0)	0.97 (1.71)	1 (0.25∼2)	−0.93 (2.23)	−2 (−3 ∼ 1)
Questionnaire – S3	−1.13 (1.72)	−2 (−2.75 ∼ 0.75)	−1.03 (1.94)	−2 (−3 ∼ 1)	−0.87 (1.89)	−1 (−2 ∼ 1)	−1.07 (2.02)	−2 (−3 ∼ 0.75)
Questionnaire – S4	−1.27 (1.84)	−2 (−3 ∼ 0)	−1.23 (1.74)	−2 (−3 ∼ 0)	−1.33 (1.79)	−2 (−3 ∼ 0)	−1.47 (1.72)	−2 (−3 ∼ 0)
Questionnaire – S5	2.2 (1.21)	2.5 (2–3)	−2.07 (1.55)	−3 (−3 ∼ −2)	2.37 (0.96)	3 (2∼3)	−2.23 (1.41)	−3 (−3 ∼ −2)
Questionnaire – S6	0.4 (1.87)	1 (−1 ∼ 2)	−1.87 (1.53)	−2.5 (−3 ∼ −1)	1.77 (1.25)	2 (1∼3)	−1.67 (1.79)	−3 (−3 ∼ 0)
Questionnaire – S7	−0.8 (1.73)	−1 (−2 ∼ 0.75)	−0.93 (1.89)	−1.5 (−3 ∼ 0.75)	−0.87 (1.7)	−1.5 (−2 ∼ 1)	−1.47 (1.85)	−2 (−3 ∼ 0)
Questionnaire – S8	−1.53 (1.85)	−2.5 (−3 ∼ 0)	−1.93 (1.39)	−2.5 (−3 ∼ −1)	−1.87 (1.31)	−2 (−3 ∼ −1)	−1.63 (1.87)	−2 (−3 ∼ −1)
Illusion onset (s)	18.36 (9.68)	21.49 (9.41–25.77)	NA	NA	14.57 (12.14)	13.75 (5.17∼18.85)	NA	NA
Proprioceptive drift (mm)	19.6 (21.82)	18.33 (5 ∼ 33.33)	9.54 (18.53)	8.33 (−5 ∼ 25)	9.94 (23.49)	11.67 (−3.33 ∼ 25)	−2.07 (14.03)	−5 (−13.33∼10)
Nociceptive drift (mm)	8.45 (17.96)	5 (−1.67 ∼ 18.33)	−2.59 (13.13)	−1.67 (−11.67∼1.67)	5.06 (19.18)	1.67 (−6.67 ∼ 11.67)	−10.11 (20.47)	−11.67 (−18.33∼1.67)

SD, standard deviation; 1Q ∼ 3Q, first to third quartile.

The table displays summary statistics for each experimental variable. In Experiment 1 (Questionnaire), data were analyzed from 30 participants. In Experiment 1 (illusion onset), data were analyzed from 18 participants in the VT_Con_ condition and 15 participants in the VN_Con_ condition. Experiment 2 (proprioceptive drift) and Experiment 3 (nociceptive drift) each included data from 29 participants.

Finally, we observed a statistically significant positive Pearson correlation between the illusion-related proprioceptive drift in the tactile RHI and N-RHI, i.e., *VT*_*Con*_ minus *VT*_*Inc*_ correlated with *VN*_*Con*_ minus *VN*_*Inc*_ (*r* (27) = 0.50, 95% CI = [0.16; 0.73], *p* = 0.006, BF_10_ = 9.95) (see Figure S1). Thus, across individuals, the degree of proprioceptive drift in one illusion predicted proprioceptive drift in the other illusion. See Figure S1.

#### VAS results

VAS ratings were significantly higher in the VN_Con_ condition as compared to the VN_Inc_ condition (*t(28)* = 3.19, 95%CI = [1.2, 5.52], *p* = 0.003, *d*_*z*_ = 0.59). On the contrary, VAS ratings did not differ significantly across blocks or sexes (all *p* > 0.06). See Table S2.

### Experiment 3

#### Nociceptive drift results

**Figure 5:**
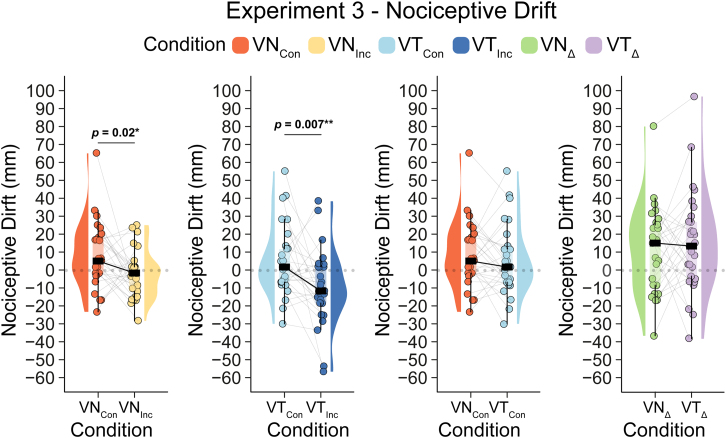
Nociceptive drift in experiment 3 Raincloud plots illustrate the individual data points and distributions for the key condition comparisons. Data are represented as medians and IQR. Note: *p* < 0.05 (∗), *p* < 0.01 (∗∗), *p* < 0.001 (∗∗∗); reported *p*-values are uncorrected. Δ denotes the difference between congruent and incongruent conditions.

One participant was determined to be an outlier in all 4 conditions and removed from further analysis, leaving 29 datasets. After the removal of the outlier, the data were normally distributed (Shapiro‒Wilk *p value* in each condition >0.08) and met the assumption of homogeneity of variance (Levene’s test *p value* = 0.57). Thus, we performed a 2 × 2 repeated-measures ANOVA, which revealed a significant main effect of congruence (*F*(1,28) = 11.619, *p* = 0.002, *η*_*p*_^2^ = 0.293) but no main effect of modality (*F*(1,28) = 3.577, *p* = 0.069, *η*_*p*_^2^ = 0.113) or interaction effect (*F*(1,28) = 0.531, *p* = 0.472, *η*_*p*_^2^ = 0.019). Thus, as hypothesized, multisensory congruence had a critical effect on nociceptive drift. See Figure 5.

Consistent with the significant main effect of congruence and our hypothesis, the planned pairwise comparisons revealed that nociceptive drift toward the rubber hand was significantly greater after *VT*_*Con*_ (M = 5.06, SD = 19.18) than after *VT*_*Inc*_ (VT_Inc_: M = −10.11, SD = ±20.47) (*t(28)* = 2.94, 95% CI = [4.59, 25.75], *p* = 0.007, BF_10_ = 6.52, *d*_*z*_ = 0.55) and after *VN*_*Con*_ (M = 8.45, SD = ±17.96) than after *VN*_*Inc*_ (*VN*_*Inc*_: M = −2.59, SD = 13.13) (*t(28)* = 2.53, 95% CI = [2.11, 19.96], *p* = 0.02, BF_10_ = 2.89, *d*_*z*_ = 0.47). Thus, both variants of the RHI were associated with a significant shift in pain localization toward the rubber hand. See Figure 5.

The lack of a significant interaction effect and a significant main effect of modality argues against substantial differences in nociceptive drift between the two variants of the RHI. This assessment was further underscored in the post hoc pairwise tests, which were performed for purely descriptive purposes: the nociceptive drift in *VT*_*Con*_ and *VN*_*Con*_ (*t(28)* = 0.91, 95% CI = [−4.24, 11.02], *p* = 0.37, BF_01_ = 3.47, *d*_*z*_ = 0.17) and the difference between *VN*_*Inc*_ and *VT*_*Inc*_ (*t(28)* = 1.73, 95% CI = [−1.38, 16.43], *p* = 0.09, BF_01_ = 1.35, *d*_*z*_ = 0.32) were not significantly different.

In a post hoc exploratory analysis, we found no statistically significant correlation between the congruence-specific nociceptive drift in the visuotactile and visual nociceptive illusions (i.e., *VT*_*Con*_ minus *VT*_*Inc*_ did not correlate with *VN*_*Con*_ minus *VN*_*Inc*_) (*r* (27) = 0.30, 95% CI = [−0.08; 0.6], *p* = 0.116, BF_01_ = 0.87). See Figure S1.

#### VAS results

VAS ratings did not differ significantly across conditions or sexes (all *p* > 0.38). On the contrary, pain in block 1 was significantly lower as compared to pain in block 2 (*V* = 109, ^*95%*^CI = [−7.7, −0.25], *p* = 0.03, *r*_*C*_ = −0.46). Finally, the felt pain in Block 3 was significantly higher as compared to the pain felt in Block 6 (*V* = 317.5, ^*95%*^CI = [0.4, 6.9], *p* = 0.03, *r*_*C*_ = 0.46). See Table S3.

## Discussion

In this study, we (1) compared the RHIs induced by touch (tactile RHI) and nociceptive stimulation (N-RHI) in the same experiments to assess the specific effects of multisensory congruence versus incongruence in the two illusion variants and (2) examined the spatial updating of pain localization following both types of RHI using a novel nociceptive drift task. The two key findings were as follows. First, the N-RHI and tactile RHI produced similarly strong multisensory congruence illusion effects, as evidenced by the questionnaire ratings (experiment 1) and the proprioceptive drift (experiment 2). This finding indicates that the N-RHI operates through multisensory congruence mechanisms similar to those of the tactile RHI. Second, both variants of the RHI were associated with significant nociceptive drift, in which the perceived location of the painful stimulus shifted toward the rubber hand, with similar magnitudes of drift for both illusions. This finding indicates that the sense of body ownership is linked to the spatial alignment of visual and pain representations, similar to the well-established recalibration of vision and proprioception. These findings suggest that pain is encoded in external spatial reference frames, similar to other somatosensory modalities, and follows the principle of maximizing perceptual coherence and minimizing intersensory mismatch, highlighting the spatial and multisensory nature of pain processing.

In the first experiment, participants’ responses to the hand ownership statements (S1 and S2) were significantly greater after the tactile RHI than after the N-RHI when we directly compared the two congruent illusion conditions (*VT*_*Con*_ vs. *VN*_*Con*_). This result could indicate that the tactile illusion was stronger, but it may also reflect a nonspecific effect related to the stimulus modality (laser versus cotton swab). Notably, we did not observe a significant difference in illusory hand ownership ratings when the control conditions were analyzed, and the difference scores were compared across illusions, i.e., (*VN*_*Con*_ minus *VN*_*Inc*_) versus (*VT*_*Con*_ minus *VT*_*Inc*_). The lack of a significant illusion congruence effect in the subjective reports was mirrored in the proprioceptive and nociceptive drift data, with no significant interaction effects observed in the 2 × 2 ANOVAs. However, we observed an unexpected main effect of modality for proprioceptive drift, with the N-RHI being associated with greater proprioceptive drift regardless of condition (congruent or incongruent), which is the opposite pattern of that observed in the hand illusion ratings for *VT*_*Con*_ and *VN*_*Con*_, as mentioned earlier. Thus, considering all findings from the first two experiments, we conclude that the tactile RHI and N-RHI produce similar illusion effects, as the strongest evidence points in this direction.

The similarly strong congruence-specific illusion effects in questionnaire ratings and proprioceptive drift suggest that both illusions operate under the same multisensory congruence rule. The current results, together with our previous study,^22^ emphasize the importance of nociceptive information in multisensory correlations that drive changes in body ownership. This finding is theoretically significant, suggesting that pain processing plays an important role in the multisensory model of body ownership.^1^^,^^4^^,^^6^^,^^10^^,^^35^ However, we should clarify that, on the basis of the current data, we cannot conclude that nociceptive and tactile inputs are *weighted* equally in the multisensory integration process of body ownership since we did not investigate trimodal (visual-tactile-nociceptive) conditions while systematically varying congruence and incongruence for each pair of sensory modalities simultaneously to determine whether visuotactile congruence ‘dominates’ over visual nociceptive congruence or vice versa.

The onset times for the two illusions were also comparable, with the onset of the tactile RHI being approximately 15 s (*n* = 18) and that of the N-RHI being approximately 18 s (*n* = 15). These onset times correspond to the delivery of approximately 6–7 bisensory stimuli (either visuo-tactile or visuo-nociceptive), which is consistent with the number of stimuli needed to elicit the illusion in previous studies. However, prior studies often used a higher stimulus delivery rate than that used in the current study, resulting in shorter onset times.^4^^,^^10^^,^^36^^,^^37^ The onset time reflects the gradual accumulation of multisensory evidence over repeated visual and somatosensory stimulations, driving the emergence of the illusion.^38^ Thus, similar onset times are consistent with the involvement of similar multisensory evidence accumulation processes. However, we observed significantly faster onset times for the tactile RHI (M = 10.66 vs. M = 19.39 for the N-RHI) when analyzing the data from the subset of 10 individuals who experienced *both* illusions. This result suggests that the two bimodal combinations may differ in their efficacy in driving the emergence of the illusion. However, this observation should be interpreted with caution, as it is based on an exploratory post hoc analysis in a small subsample.

The second major finding of our study was that the spatial localization of pain shifts toward the rubber hand in both the nociceptive and tactile variants of the RHI. This shift was demonstrated in an experiment introducing a novel behavioral test for the RHI, the “nociceptive drift test”, inspired by the classic proprioceptive drift task. In this task, participants, with their eyes closed, were asked to indicate the perceived location of a painful stimulus delivered to their hidden real hand before and after being exposed to a trial of congruent or incongruent visual nociceptive or visuotactile RHI stimulation. We found that the location of painful laser stimulation was perceived as being closer to the rubber hand following both congruent visual nociceptive stimulation and congruent visuotactile stimulation than after the corresponding incongruent control condition. This finding shows that the RHI is associated with spatial recalibration of nociception and vision, just as the illusion is associated with a similar recalibration of proprioception and vision. Since recalibration of vision and proprioception serves to ensure matching spatial representations of the senses in the external space (peripersonal space) and minimize intersensory spatial conflict, the presence of nociceptive drift suggests that pain is also remapped in external spatial coordinates and that it follows the same policy of maximizing perceptual coherence and minimizing intersensory mismatch. This phenomenon is expected because, to protect the body from potential and actual tissue damage, spatial pain representations must be accurate and aligned with multisensory body representations. Our nociceptive drift results were also consistent with the questionnaire findings from experiment 1, in which participants affirmed the questionnaire statement of sensing pain from the location where they saw visual stimulation (S5 and S6). Thus, both the questionnaire results and the nociceptive drift findings indicate that the pain experience shifts toward the fake hand during the RHI. These observations underscore the spatial, dynamic, and multisensory nature of pain.

From a theoretical perspective, our findings contribute to discussions on the role of information carried by small-diameter (C and Aδ) primary afferent fibers in body ownership.^39^^,^^40^^,^^41^ The signals from C and Aδ fibers have drawn significant interest because they innervate all tissues of the body and carry information about the body’s physiological state, capturing affective‒motivational dimensions of bodily feeling, including not only pain but also temperature, itch, sensual touch, muscular and visceral sensations, vasomotor activity, hunger, thirst, and air hunger.^42^ However, demonstrating the contribution of C and Aδ signals to body ownership has been challenging, as these signals are often “contaminated” by tactile inputs from mechanoreceptors when brushes, probes, objects, or thermal stimulators interact with the skin.^14^^,^^16^^,^^43^^,^^44^^,^^45^ Additionally, sensations from internal organs, such as heartbeats or bladder tension, involve signals from small-fiber C and Aδ afferents mixed with other signals from mechanoreceptors and proprioceptors. Therefore, from this physiological perspective, the findings from the current experiments and our previous study^22^ are valuable, as they provide a compelling example of a specific contribution of C and Aδ signals to body ownership since the Nd:YAP laser selectively activates C and Aδ signals in the skin without activating mechanoreceptors (or proprioceptors).^23^^,^^46^^,^^47^^,^^48^^,^^49^^,^^50^ In the current study, we investigated only nociception, so future research is needed to examine the hypothesis that our principal observations can be generalized to other C and Aδ fiber-mediated senses, such as thermosensation.

In conclusion, this study demonstrated that congruent versus incongruent visual nociceptive and visuotactile stimulation elicit similarly strong RHI effects and that both the N-RHI and the tactile RHI are associated with a shift in the spatial localization of pain toward the rubber hand—an effect we termed “nociceptive drift.” Although it may seem counterintuitive that pain can be experienced outside the physical body in a fake hand, our findings highlight that pain is an integral part of the multisensory experience of one’s body. This experience is dynamically and continuously updated, so when the fake hand is perceived as one’s own in the RHI, the fake hand also becomes the source of the painful sensations triggered by laser-activated nociceptors in the skin of the real hand. This multisensory perspective on pain may have important theoretical implications for clinical pain research, particularly given the frequent co-occurrence of pain with changes in bodily awareness and body representation in various pain disorders.^18^^,^^20^^,^^51^

### Possible clinical implications

Although our findings are based on healthy participants, they may have implications for research into various pain disorders.^18^^,^^20^^,^^51^^,^^52^^,^^53^ Our results suggest that the nociceptive system contributes to body ownership and that nociceptive signals integrate with other sensory inputs according to multisensory integration principles. Future research could explore whether modulating multisensory integration in N-RHI paradigms can help reduce pain in chronic pain patients, such as those with fibromyalgia^54^^,^^55^ or complex regional pain syndrome (CRPS)^56^ (for a review on the use of bodily illusions in clinical pain modulation strategies, see Moseley et al.^51^). Since these conditions affect body representation,^57^^,^^58^^,^^59^^,^^60^^,^^61^ it is reasonable to hypothesize that altering body representations in these clinical populations might also modulate pain. This technique warrants further exploration as a potential avenue for new pain treatment strategies. Moreover, the N-RHI could serve as a valuable tool for investigating how nociceptive inputs integrate with other body-related sensory signals in individuals with a disturbed sense of limb ownership after stroke.^62^^,^^63^ This approach would allow researchers to determine whether nociceptive input integrates with other sensory modalities, as in healthy individuals, or whether nociceptive integration and processing are altered in these individuals. These alterations are consistent with recent findings linking poststroke body ownership disruptions to changes in thermosensation, another C-fiber-mediated modality.^62^ In addition, pain insensitivity is well-documented in patients with self-injury behavior^64^ and has together with feelings of dissociation been suggested as a risk factor for developing self-injury. The current study points to a possible new direction of therapy where multisensory training may reduce the frequency and gravity of self-injury by normalizing the link between body ownership and nociception.

### Limitations of the study

Several limitations of this study should be acknowledged. First, it is difficult to control for differences in attentional responses when nociceptive and tactile stimulation are compared, so we cannot exclude the possibility that differences in attention may have influenced our results. When a body part is exposed to a noxious stimulus, our attention tends to be automatically turned toward that part to initiate protective measures,^18^ and attention is a cognitive function linked to pain.^65^^,^^66^^,^^67^^,^^68^^,^^69^ However, the similar strengths of the RHIs observed in the nociceptive and tactile conditions may suggest that the engagement of attention was not substantially different between the two illusions. In addition, the level of pain was moderate, which was matched in the incongruent control condition, and the experiments were long, meaning that the participants likely acclimated to the delivery of noxious stimulation, resulting in less attention. Second, the nociceptive and tactile stimuli were matched as closely as possible, including the same number of stimuli per trial (*n* = 40), the same diameter of tactile and laser stimuli (7 mm), the same frequency of stimuli (0.33 Hz), and the same duration of visual stimuli (130 ms), we could not directly match the intensity of the nociceptive laser (measured in mJ/mm^2^) with the intensity of the tactile touches applied using cotton swabs (measured in newtons). Furthermore, although the durations of the two types of somatosensory stimuli were similar, the laser machine provided a laser pulse that lasted exactly 7 ms, whereas the tactile cotton swabs were manually delivered, resulting in more variability and longer skin contact times (approximately 130 ms). Thus, we cannot exclude the possibility that differences in stimulus intensity and stimulus duration may have influenced the results. Third, tactile stimulation may have had a clearer offset perceptually than Nd:YAP laser pain stimulation did because of physiological aspects of the nociceptive system, which results in a prolonged “burning” sensation (C fibers)^70^^,^^71^^,^^72^ after the initial “pinpricking” sensation (Aδ fibers). Thus, we cannot exclude the possibility that those differences in temporal precision in the tactile and nociceptive offset signals influenced our results. Finally, for future investigations, it may be valuable to include sex-balanced samples and physiological indicators of autonomic nervous system activity, e.g., the skin conductance response, and compare these indicators across the two variants of the illusion.^73^^,^^74^ However, we did not include physiological measures in the current study because nociceptive stimuli can modulate autonomic nervous system reactivity, making it difficult to isolate RHI-related effects from broader, unspecific modulations.

## Resource availability

### Lead contact

Further information and requests for resources should be directed to and will be fulfilled by the lead contact, Sara Coppi (sara.coppi@ki.se).

### Materials availability

No new materials (i.e., reagents) were created in the process of this human study.

Additional analyses of the results are provided in the supplemental information.

### Data and code availability

• Pseudonymized source data (questionnaire ratings, proprioceptive drift, and VAS data) have been deposited at OSF (https://osf.io/vfwge/?view_only=f5064f0e2a644f2483660af7b82c23ec) and are publicly available as of the date of publication.
• The R code used for data analysis has been deposited at OSF (https://osf.io/vfwge/?view_only=f5064f0e2a644f2483660af7b82c23ec).
• Any additional information required to reanalyze the data reported in this paper is available from the lead contact upon request.

## Acknowledgments

This project was funded by the Horizon 2020 European Research Council (Advanced Grant SELF-UNITY #787386), the 10.13039/501100004359Swedish Research Council (# 2017-03135), the 10.13039/100007464Torsten Söderberg Foundation, and 10.13039/501100003792Hjärnfonden.

The authors thank Martti Mercurio for developing the program in C++ to control the Nd:YAP laser. The authors acknowledge the professional illustrator Mattias Karlén for providing Figure 1.

## Author contributions

S.C. and H.H.E. designed the study. S.C. collected and analyzed the data. S.C. and H.H.E. wrote the manuscript. S.C., H.H.E., and K.B.J. provided revisions and approved the final version of the manuscript for submission.

## Declaration of interests

The authors declare that they have no conflicts of interest, financial or otherwise.

## STAR★Methods

### Key resources table

**Table:** 

REAGENT or RESOURCE	SOURCE	IDENTIFIER
**Deposited data**		

Pseudonymized source data	This paper	https://osf.io/vfwge/?view_only=f5064f0e2a644f2483660af7b82c23ec
R code	This paper	https://osf.io/vfwge/?view_only=f5064f0e2a644f2483660af7b82c23ec

**Software and algorithms**		

RStudio	R Core Team^75^	https://www.R-project.org/

### Experimental model and study participant details

#### Participants

Thirty naive healthy participants were tested per experiment (see Table 1 for all participant details, total *n* = 90–51 F), with different participants recruited for each experiment – Experiment 1: *N* = 30(8 M–22 F); Experiment 2: *N* = 30(16 M–14 F); Experiment 3: *N* = 30(15 M–15 F). All participants within the same experiment completed every experimental condition as part of a within-subjects design. Therefore, no group allocation was necessary. No formal power analysis was conducted before the study commenced since the current study investigates novel questions and uses a novel measure. Instead, the sample size (*N* = 30) was based on previous literature on rubber hand illusions and pain stimulation.^16^^,^^22^ A sample size of 30 usually provides sufficient statistical power in behavioral studies on the rubber hand illusion, and we reasoned that it should be appropriate for detecting psychologically meaningful effects. The sample size was determined before the study started. We chose to have three separate experiments to keep participants as naive as possible for each outcome measure (e.g., the participants reporting hand location in the proprioceptive drift task had not seen any questionnaire statements about possible subjective experiences of the illusion).

The participants needed to be fully healthy; had no history of skin disease or strong skin reaction after 30 min of sunbathing; had not taken any medications (including painkillers), recreational drugs or alcohol within the 24 h prior to the experiment; were between 18 and 50 years old; had not been exposed to the solarium, sunbathing or nail treatment with UV lights for 3 weeks before the experiment; and were naiive to the purpose of the study. Additionally, we excluded *a priori* volunteers who had previously taken part in bodily illusion experiments, such as rubber hand illusion, full-body illusion and virtual reality bodily illusion experiments. Handedness was assessed at the beginning of each experiment using the Edinburgh Handedness Inventory.^76^

The Swedish Ethics Review Authority approved all the experiments. Prior to the experiment, each participant provided written informed consent. After the experiment, the participants received compensation for their participation (either a cinema ticket or a small sum of money).

### Method details

#### Procedure

Participants who met the inclusion and not the exclusion criteria (see above) came to the laboratory for a unique session of either 60 min (Experiment 1) or 115 min (Experiments 2 or 3). Once the participants provided their written informed consent, the experiment began. At the beginning of the session, we calibrated the nociceptive laser intensity so that the participants could feel pain scored at approximately 20–30 on a visual analog scale (VAS), ranging from 0 mm (‘no pain’) to 100 mm (‘the worst imaginable pain’).^77^ The detailed calibration procedure is described elsewhere.^22^ After calibration, the experimental rubber hand illusion session started (see further details below). During the entire experiment, participants wore headphones (binaural ATH-M40x professional monitor headphones, Audio-technica U.S., Inc.) and listened to white noise (right ear, 50.7 dBA; left ear, 51.5 dBA, as measured via Mini Sound Level Meter, Model ST-805, Clas Ohlson AB) to avoid hearing the ‘beep’ produced by the laser machine. Additionally, to protect the eyes from potential accidental damage, both the participant and the experimenter wore special protective glasses (000-G0140-RETR-21, PROTECT Laserschutz GmbH) during the entire session.

#### Tactile rubber hand illusion setup

The rubber hand illusion setup was adapted from the original study of Botvinick and Cohen.^5^ The participant was seated in front of a table and asked to put their left hand on the table in a relaxed posture next to a prosthetic hand glove filled with gypsum (“the rubber hand”). As in Botvinick and Cohen’s original report, we used a left rubber hand. Research over the past decades has shown that the RHI is effective for both left and right rubber hands in groups of healthy individuals who are predominantly right-handed, with little evidence for significant differences in illusion strength between left and right RHIs.^78^^,^^79^ Moreover, we reasoned that using a left rubber hand might be more clinically relevant, as disturbances in the sense of body ownership after right-hemisphere strokes (e.g., asomatognosia and somatoparaphrenia) typically involve the upper left limb.^80^^,^^81^^,^^82^ The participant’s real left hand was hidden from their view behind a screen while the rubber hand was fully visible. A black cloth was used to cover the spatial gap between the participants’ left shoulder and the rubber hand so that it appeared visually to the participant that the rubber hand may be their own hand. For visuotactile stimulation, we used cotton swabs to simultaneously touch the participant’s hidden real left hand and the rubber hand; tactile stimulation activated mechanoreceptors in the skin. The experimenter held the cotton swabs and briskly and briefly tapped the skin at a single point (no stroking). We chose cotton swabs because their diameter is ∼7 mm, matching the diameter of the laser used in nociceptive laser stimulation under some of the experimental conditions (see further below). In each block of tactile stimulation, 40 cotton-swab stimulations were manually delivered by a trained experimenter (SC) over 2 min (0.33 Hz), and the duration of each stimulus was ∼130 ms (as estimated in separate experiments with a high-speed camera). The area of stimulation was the dorsum of the left hand within dermatomes C6 and C7. In VT_Con_, corresponding stimulation sites on the rubber hand and real hand were simulated synchronously; in VT_Inc_, nonmatching sites were asynchronously stimulated with a 1-s delay.

#### Nociceptive rubber hand illusion setup

The N-RHI setup and protocol were the same as those in.^22^ The setup was the same as that used in the tactile RHI described above, except for the visual and somatosensory stimuli. In this experiment, we used a nociceptive stimulus instead of a tactile stimulus, which was delivered to the dorsum of the left hand with a Nd:YAP laser (Stimul 1340 Neurolas, Deka, Calenzano, Italy) operating at a wavelength of 1.34 μm. This laser has the capacity to selectively activate C and Aδ fibers^23^^,^^24^ without activating Aβ-fiber mechanoreceptors.^25^ The chosen diameter was 7 mm, and the length of the stimulus was 7 ms. We delivered 40 stimuli per block with a pace of 0.33 Hz on dermatomes C6 and C7 of the dorsum of the left hand. As a visual stimulus, we delivered a nonnociceptive red laser light stimulus with a diameter of 7 mm and a length of 130 ms with a diode laser (VLM-650-01 PT, Laser Diode 650 nm, 1 MW, 10.4 mm DIA). This visual stimulus was delivered to the rubber hand at corresponding sites in VN_Con_ and noncorresponding sites in VN_Inc._ In VN_Con_, the visual and nociceptive stimuli were delivered synchronously; in VN_Inc_, the stimuli were delivered asynchronously with a delay of 1 s. Additionally, the visual stimuli were provided with a delay of 60 ms compared with the nociceptive input, which is consistent with previous studies that reported that this delay creates the impression of simultaneity between visual and nociceptive input.^83^^,^^84^^,^^85^ Both lasers were controlled via a program developed in the software C++.

### Quantification and statistical analysis

#### Experiment 1 – RHI questionnaire

After each block, the participants were given a questionnaire consisting of 8 statements (see Table 2). In the questionnaire, we included 4 illusion-related statements (S1, S2, S5, and S6). S1 and S2 assessed the feeling of ownership of the rubber hand, whereas S5 and S6 assessed the referral of touch or pain to the rubber hand. Additionally, we included 4 control statements (S3, S4, S7, and S8) to test for unspecific cognitive bias, task compliance, and confabulation. The statements were presented on cards, and the participants verbally reported their agreement or disagreement with each statement on a 7-point Likert scale from −3 (strongly disagree) to +3 (strongly agree). The experimenter directly noted these responses in an Excel sheet. The statements were presented in a randomized order, and each block featured a different randomization of the statement order.

#### Experiment 1 – Illusion onset

If participants reported a score ≥1 for the experimental statement S1 (“*It felt as if the rubber hand was my own*”) after the experimental conditions *VT*_*Con*_ and/or *VN*_*Con*_, then at the end of the experiment, we repeated that condition an additional three times to measure the illusion onset, defined as the duration of repeated visuotactile or visual nociceptive stimulation before a noticeable illusion was experienced.^4^^,^^36^ During this measurement, the participants pressed a button on a number-pad keyboard with their right hand when the illusion started to occur, i.e., when they started to feel like the rubber hand was their own hand. The instructions were provided verbally as follows: *‘Please press the button as soon as you feel as if the rubber hand is your own’*. The measurements were automatically recorded on the same computer used to deliver the laser stimulations. We averaged the three illusion onset reports to obtain a more precise estimate of this outcome measure for each participant.

#### Experiment 2 – Proprioceptive drift

During the proprioceptive drift task, the participants were asked to close their eyes, place their right finger above a ruler and slide it up to where they felt the location of their left index finger. The ruler was placed 8.5 cm above the two left hands so that the participants would not encounter any obstacles or tactile reference points while sliding. Each condition was tested three times, and the proprioceptive drift was measured before (baseline) and after the visuotactile or visual nociceptive stimulation condition in each block. The proprioceptive drift was calculated as the difference between the measures obtained before and after each block. A positive change demonstrated a shift in hand position toward the rubber hand.^5^^,^^28^^,^^86^^,^^87^ To improve the accuracy of this indicator, we averaged the three measures for each participant within each condition. The initial position of the right index finger on the ruler was randomized to avoid participants learning a specific movement response. We trained the participants to perform the task correctly, with two practice trials conducted before the actual experiment started.

#### Experiment 3 – Nociceptive drift

The nociceptive drift test procedure was identical to the proprioceptive drift test described above with two critical changes. Before and after the presentation of each visuotactile or visual nociceptive rubber hand condition, a single nociceptive laser stimulus was delivered on the first knuckle of the index finger of the participant’s (unseen) real left hand. Directly after this nociceptive stimulus, the participants were asked ‘*please indicate where you felt the pain’*.

#### All experiments – Pain visual analog scale (VAS) ratings

We assessed the perceived pain intensity after each delivered block to monitor sensitization and/or habituation continuously. To register the participants’ pain responses, we adapted a variable assessment transducer (TSD115, Biopac Systems, Inc.) to create a 100 mm VAS slider. The VAS device (TSD 115) was linked to BIOPAC hardware (MP 160, BIOPAC Systems, Inc.), and the responses were visualized by the experimenter using AcqKnowledge software (BIOPAC Systems, Inc.). The VAS slider was labeled at the extreme left with ‘no pain’ (equal to 0 mm) and at the extreme right with ‘the worst imaginable pain’ (equal to 100 mm).

The experimenter instructed the participants after each delivered block with the following sentence: “*You should slide on this slider to indicate the amount of pain you felt. Bear in mind that the starting position ‘no pain’ means that you did not feel any pain and that, as soon as you move the slider, it means that you can discriminate the pain from other sensations. You should move the slider only if you felt pain, since it is a pain scale*”.

#### Data analysis

We analyzed the data using the statistical software RStudio 3.6.1.^75^ and set the significance threshold alpha at 5% (*α* = 0.05). An outlier check identified one outlier each in Experiment 1 (illusion onset), Experiment 2 (proprioceptive drift) and Experiment 3 (nociceptive drift). Outliers were defined as responses that deviated by ±3 standard deviations (SDs) from the mean in at least one of the 4 conditions (i.e., VN_Con_, VT_Con_, VN_Inc_, and VT_Inc_). We did not check for outliers in Experiment 1 (RHI Questionnaire) because the outcome variable was a bounded Likert scale (−3 to +3), where high or low values indicated strong agreement or disagreement with the statement. After outlier exclusion, the sample sizes were as follows: 30 participants for Experiment 1 (RHI Questionnaire), 18 participants for Experiment 1 (illusion onset, visuotactile condition), 15 participants for Experiment 1 (illusion onset, visual nociceptive condition), 29 participants for Experiment 2 (proprioceptive drift), and 29 participants for Experiment 3 (nociceptive drift).

A 2 × 2 repeated-measures ANOVA was conducted for continuous data from Experiments 2 (proprioceptive drift) and 3 (nociceptive drift) meeting the assumptions of normality, homogeneity of variance, continuity of data, and absence of outliers. The Shapiro‒Wilk test was used to assess normality, whereas Levene’s test was used to evaluate homogeneity of variance. Since Experiment 1 utilized a Likert scale, the data were ordinal and noncontinuous. Therefore, we did not conduct a standard 2 × 2 ANOVA, as one of its key assumptions is the presence of continuous data. Instead, we employed nonparametric tests, such as the Wilcoxon signed-rank test, for within-subject comparisons.

We also performed planned pairwise comparisons in all the experiments. Since these comparisons were planned *a priori*, full post hoc correction was not necessary. Additionally, we did not correct for multiple comparisons in the planned pairwise tests, nor did we apply such correction for post-hoc tests, which were reported purely for descriptive purposes. For continuous data (Experiment 1 – illusion onset; Experiment 2 – proprioceptive drift; and Experiment 3 – nociceptive drift), we applied the Shapiro‒Wilk test to assess normality. If the *p value* was >.05, we used parametric tests such as t tests; if the *p value* was <.05, we used nonparametric tests such as the Wilcoxon signed-rank test (within-subjects comparison) and the Wilcoxon sum‒rank test (between-subjects comparison). Similarly, we reported the Pearson correlation when parametric data were analyzed and the Spearman correlation when nonparametric data were analyzed. All tests were two-tailed.

We also conducted Bayesian analysis using the default priors in the R package BayesFactor^88^ and reported Bayes factors (BFs) (i.e., ${BF}_{10}=\frac{P\left(D|H_{1}\right)}{P\left(D|H_{0}\right)}$ when the evidence is in favor of the alternative hypothesis or ${BF}_{01}=\frac{P\left(D|H_{0}\right)}{P\left(D|H_{1}\right)}$ when the evidence is in favor of the null hypothesis). Additionally, we reported the effect sizes of the analyses. *Cohen’s d*_*z*_ was reported when paired t tests were performed,^89^ the paired rank-biserial correlation (*r*_*C*_,^90^^,^^91^) was reported when nonparametric Wilcoxon tests were performed, and the *Glass biserial correlation coefficient* (*r*_*G*_)^91^ was reported when nonpaired tests were performed.
