# Supplementary material for: Feeling pain from a rubber hand: Nociceptive drift in the rubber hand illusion
Source: iScience. 2025 May 19;28(6):112690. doi: 10.1016/j.isci.2025.112690 (PMC12178805; doi:10.1016/j.isci.2025.112690)
Supplement: Document S1. Figure S1 and Tables S1–S3 [file mmc1.pdf]

iScience, Volume *28*

Supplemental information

Feeling pain from a rubber hand: Nociceptive drift in the rubber hand illusion

Sara Coppi, Karin B. Jensen, and H. Henrik Ehrsson

Supplemental figures

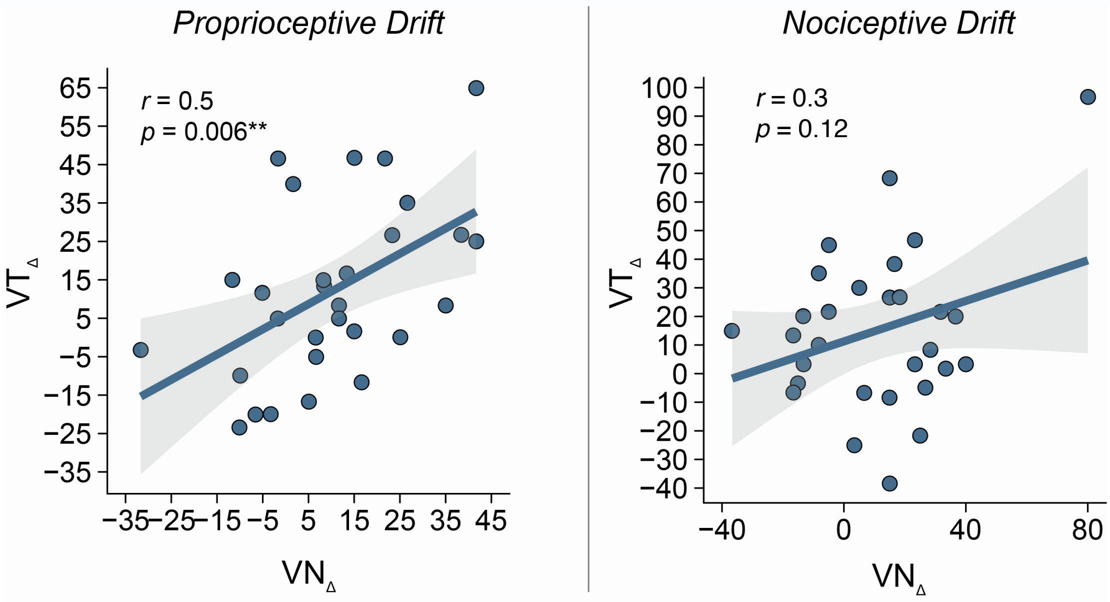

***Figure S1*. *Correlations, related to Figures 4 and 5*.** *The figures display linear regression plots illustrating the correlational analyses for Experiment 2 (Proprioceptive Drift) and Experiment 3 (Nociceptive Drift).*
*Note: * = p < 0.05, ** = p < 0.01, *** = p < 0.001; p-values are uncorrected. Δ indicates the difference between congruent and incongruent conditions.*

Supplemental tables

Variable	*Level*	*Mean (SD)*	*Median (1Q~3Q)*
**Condition**	VN_{Con}	28.73 (9.97)	27.45 (22.45~32.95)
	VN_{Inc}	26.34 (11.14)	25.95 (17.42~34.7)
**Gender**	*Female*	26.27 (9)	26.75 (20.59~29.97)
	Male	31.02 (10.23)	29.58 (22.08~36.69)
**Block**	*Block 1*	28.07 (10.09)	28.55 (22.75~34.7)
	Block 2	27 (11.14)	25.15 (21.08~33.1)

Table S1*. *VAS descriptive statistics, experiment 1 – RHI Questionnaire, related to VAS results. *Descriptive statistics for the VAS scores from Experiment 1 are presented. SD = standard deviation; 1Q–3Q = first to third quartile range.*
No significant difference was found in VAS pain ratings between VN_{Con} *and* VN_{Inc} *conditions* ($t(29) = 1.358$, $^{95\%}CI = [-1.211; 5.998]$, $p = 0.185$, $d_z = 0.248$)*, nor between blocks* ($t(29) = 0.594$, $^{95\%}CI = [-2.622; 4.768]$, $p = 0.557$, $d_z = 0.108$) *nor between sexes* ($t(28) = 1.24$, $^{95\%}CI = [-3.13; 12.64]$, $p = 0.23$, $d_s = 0.51$).

Variable	*Level*	*Mean (SD)*	*Median (1Q~3Q)*
**Condition**	VN_{Con}	29.51 (8.81)	28.73 (22.77~38.13)
	VN_{Inc}	26.14 (8.81)	24.1 (20.37~34.77)
**Gender**	*Female*	26.14 (8.09)	24.91 (22.57~29.29)
	Male	29.39 (8.53)	30.05 (21.22~34.93)
**Block**	*Block 1*	26.19 (8.01)	23.8 (21.5~29)
	Block 2	25.89 (8.24)	25.5 (20.7~32.7)
	Block 3	26.98 (9.32)	27.5 (21.1~33.2)
	Block 4	28.89 (12.6)	25.7 (20.5~40.8)
	Block 5	28.56 (13.44)	24.1 (18.7~37.5)
	Block 6	30.46 (12.85)	29.4 (20.7~40.2)

Table S2*. *VAS descriptive statistics, experiment 2 – Proprioceptive Drift, related to VAS results. *Descriptive statistics for the VAS scores from Experiment 2 are presented. SD = standard deviation; 1Q–3Q = first to third quartile range The shown VAS results are after exclusion of one outlier from the proprioceptive drift experiment.*

Following the removal of an outlier from the proprioceptive drift experiment, the VAS data were analyzed and reported. A significant difference was found in VAS pain ratings between VN_{Con} *and* VN_{Inc} *conditions* ($t(28) = 3.19$, $^{95\%}CI = [1.2, 5.52]$, $p = 0.003$, $d_z = 0.59$) *with higher scores in the* VN_{Con} *condition, but no difference was found between blocks (all comparisons have a* $p > 0.06$*) nor between sexes* ($t(27) = 1.05$, $^{95\%}CI = [-3.09; 9.59]$, $p = 0.3$, $d_s = 0.39$)*.*

Variable	*Level*	*Mean (SD)*	*Median (1Q~3Q)*
**Condition**	VN_{Con}	32.5 (±7.53)	32.07 (29.13~35.53)
	VN_{Inc}	30.51 (±8.18)	29.97 (23.17~35.77)
**Gender**	*Female*	30.55 (±7.43)	29.03 (26.37~33.92)
	Male	32.4 (±7.09)	32.95 (29.31~34.99)
**Block**	*Block 1*	29.7 (±10.49)	27.5 (21.3~36.7)
	Block 2	32.31 (±9.98)	32.8 (25.5~36.8)
	Block 3	34.43 (±12.19)	31.2 (25.8~39.9)
	Block 4	31.84 (±9.91)	31.3 (23~39.3)
	Block 5	30.59 (±11.15)	31.1 (22.4~36.2)
	Block 6	30.16 (±9.15)	29.3 (23.5~35.6)

Table S3*. *VAS descriptive statistics, experiment 3 – Nociceptive Drif, related to VAS results. *Descriptive statistics for the VAS scores from Experiment 3 are presented. SD = standard deviation; 1Q–3Q = first to third quartile range. The shown VAS results are after exclusion of one outlier from the nociceptive drift experiment.*
Following the removal of an outlier from the nociceptive drift experiment, the VAS data were analyzed and reported. No significant difference was found in VAS pain ratings between VN_{Con} *and* VN_{Inc} *conditions (*$V = 241$, $^{95\%}CI = [-0.9, 4.53]$, $p = 0.4$, $r_C = 0.19$*). Only few blocks differed between each other in terms of VAS pain ratings. Pain in block 1 was significantly lower as compared to pain in block 2 (*$V = 109$, $^{95\%}CI = [-7.7, -0.25]$, $p = 0.03$, $r_C = -0.46$*). The felt pain in Block 3 was significantly higher as compared to the pain felt in Block 6 (*$V = 317.5$, $^{95\%}CI = [0.4, 6.9]$, $p = 0.03$, $r_C = 0.46$*).*
No significant difference was found between sexes ($t(27) = 0.69$, $^{95\%}CI = [-3.68, 7.4]$, $p = 0.5$, $d_s = 0.26$*).*
